# Factors of Persistent Limited Exercise Tolerance in Patients after COVID-19 with Normal Left Ventricular Ejection Fraction

**DOI:** 10.3390/biomedicines10123257

**Published:** 2022-12-15

**Authors:** Katarzyna Gryglewska-Wawrzak, Agata Sakowicz, Maciej Banach, Marek Maciejewski, Agata Bielecka-Dabrowa

**Affiliations:** 1 Department of Cardiology and Congenital Diseases of Adults, Polish Mother’s Memorial Hospital Research Institute (PMMHRI), 93338 Lodz, Poland; 2 Department of Medical Biotechnology, Medical University of Lodz, 90419 Lodz, Poland; 3 Department of Preventive Cardiology and Lipidology, Medical University of Lodz, 90419 Lodz, Poland

**Keywords:** COVID-19, exercise intolerance, global peak systolic strain, body mass compartments, personalization of body structure

## Abstract

Exercise intolerance de novo is one of the most common reported symptoms in patients recovering from the Coronavirus Disease 2019 (COVID-19). The present study determines etiological and pathophysiological factors influencing the mechanism of impaired exercise tolerance in patients during Long-COVID. Consequently, the factors affecting the percentage predicted oxygen uptake at peak exercise (%VO_2_pred) in patients after COVID-19 with a normal left ventricular ejection fraction (LVEF) were assessment. A total of 120 patients recovering from COVID-19 at three to six months after confirmed diagnosis were included. The clinical examinations, laboratory test results, echocardiography, non-invasive body mass analysis, and spiroergometry were evaluated. The subjects were divided into the following groups: study patients’ group with worsen oxygen uptake (%VO_2_pred < 80%; *n* = 47) and control group presenting%VO_2_pred ≥ 80% (*n* = 73). ClinicalTrials.gov Identifier: NCT04828629. The male gender and the percent of total body water content (TBW%) were significantly higher in the study group compared to the control group (53 vs. 29%, *p* = 0.007 and 52.67 (±6.41) vs. 49.89 (±4.59), *p* = 0.02; respectively). Patients with %VO_2_pred < 80% presented significantly lower global peak systolic strain (GLPS), tricuspid annular plane systolic excursion (TAPSE), and late diastolic filling (A) velocity (19.34 (±1.72)% vs. 20.10 (±1.35)%, *p* = 0.03; 21.86 (±4.53) vs. 24.08 (±3.20) mm, *p* = 0.002 and median 59.5 (IQR: 50.0–71.0) vs. 70.5 (IQR: 62.0–80.0) cm/s, *p* = 0.004; respectively) compared to the controls. The results of the multiple logistic regression model show that (A) velocity (OR 0.40, 95%CI: 0.17–0.95; *p* = 0.03) and male gender (OR 2.52, 95%CI: 1.07–5.91; *p* = 0.03) were independently associated with %VO_2_pred. Conclusions: Men have over twice the risk of persistent limited exercise tolerance in Long-COVID than women. The decreased (A) velocity, TAPSE, GLPS, and hydration status are connected with limited exercise tolerance after COVID-19 in patients with normal LVEF.

## 1. Introduction

Severe acute respiratory syndrome coronavirus 2 (SARS-CoV-2) causes the coronavirus disease 2019 (COVID-19). The pandemic has spread all over the world since its first appearance in Wuhan (China) in December 2019 [1]. The SARS-CoV-2 infection has a full range of clinical manifestations from asymptomatic to dyspnea, fever, fatigue, dry cough, myalgias, and chest pain, as well as imaging and laboratory abnormalities, such as bilateral ground-glass opacities on chest CT scans and lymphopenia [2]. Pneumonia, acute respiratory distress syndrome (ARDS), septic shock, and specific organ dysfunction may be other clinical presentations of COVID-19. The cardiovascular system can be also affected by COVID-19. SARS-CoV-2, through ACE2 (angiotensin-converting enzyme 2) as a receptor for the viral spike protein, can infect the heart, vascular tissues, and circulating cells [3]. This enzyme is a homologue of the angiotensin-converting enzyme (ACE) and plays an essential role in the renin–angiotensin–aldosterone system (RAAS), which involves blood pressure regulation and electrolyte homeostasis [4]. The spike protein plays a key role in the virus tissue tropism. This protein binds to the ACE2 receptor on the surface of the host cell. The binding blocks ACE2 activity and thus reduces the enzyme expression in the membrane [5]. The imbalance between ACE and ACE2 leads to an escalation in Ang II-mediated vasoconstriction and a decrease in Ang (1–7)-mediated vasodilation. Put simply, malfunctional RAAS may exaggerate the progression of COVID-19, especially during a cytokine release storm [6]. Cardiovascular cells that express ACE2 are possibly at risk for SARS-CoV-2 infection. Other factors, including expression of the host proteases that prime the virus, are required for infection as well [7]. The loss of ACE2 by SARS-CoV-2-induced internalization would be predicted to aggravate underlying cardiovascular disease (CVD) acutely and possibly in the long term. Acute cardiac injury is a common extrapulmonary manifestation of COVID-19 with possible chronic consequences. Post-acute sequelae of SARS-CoV-2 infection or long-COVID, can occur in recovering patients [8]. The clinical manifestations of cardiac involvement could range from an absolute lack of symptoms in the presence of elevated troponin levels, with or without ECG or imaging abnormalities; pulmonary embolism; acute coronary syndromes; myocarditis; acute heart failure to chronotropic incompetence; arrhythmia; and sudden cardiac death [9,10,11]. Frequent symptoms in patients who have recovered from COVID-19 also include dyspnea, fatigue, breathlessness, persistence of smell and taste disturbances, muscle aches, headaches, anxiety, sleep disturbances, and reduced exercise tolerance [12,13]. Exercise is determined by oxygen supply, oxygen uptake, and the clearance of toxic metabolites. These mechanisms are dependent on the pulmonary and cardiovascular systems to gain optimal exercise performance. Hence, there is an option to address the functional competence of the organs by coupling external adjustments to cellular respiration by studying external respiration in response to exercise [14]. The relevant tool to estimate the functional capacity during exercise is cardiopulmonary exercise testing (CPET). The main outcome of aerobic capacity is the peak oxygen uptake (VO_2_peak), defined as the maximum amount of oxygen that can be absorbed during exercise. CPET is considered the gold standard for the assessment of physical fitness and evaluating the interaction of cardiovascular, respiratory, and metabolic systems. Cardiopulmonary evaluation can be conducted safely among non-hospitalized people affected by COVID-19 symptoms. Thus, we sought to quantify and describe the extent and the main mechanisms of exercise limitation in these patients.

The aim of the study was to identify the etiological and pathophysiological factors influencing the mechanism of exercise intolerance assessed in CPET, as well as biochemical and echocardiographic parameters in the COVID-19 survivors.

## 2. Materials and Methods

### 2.1. Basic Characteristics

We recruited 120 consecutive patients from the Department of Cardiology recovering from COVID-19 at three to six months after confirmed diagnosis in this study. The subjects were hospitalized in the Department of Cardiology and Congenital Heart Diseases of Adults between December 2020 and December 2021. There were no differences in medical treatment between the groups. All patients enrolled in this study performed CPET on the ergometer. The definition of %VO_2_pred is a percentage predicted oxygen uptake at peak exercise VO_2_. The patients were divided into a group that demonstrated worse oxygen uptake (%VO_2_pred < 80%; *n* = 47) at the median age of 49 years old (median VO_2_max 17 mL/kg/min) and a control group at the median age of 55 years old who presented VO_2_pred ≥ 80% (73 patients, median 23 mL/kg/min).

The exclusion criteria were as following: diagnosis of cardiomyopathy; diagnosis of heart failure—left ventricular ejection fraction (LVEF) < 50% and signs and symptoms of heart failure or LVEF ≥ 50% with signs and symptoms and raised natriuretic peptides; unstable angina; unstable heart rhythm disorders; advanced atrioventricular block; acute pulmonary embolism; past myocardial infarction; uncontrollable arterial hypertension—systolic blood pressure ≥ 150 mmHg and/or diastolic blood pressure ≥ 100 mmHg; acute pericarditis or myocarditis; active endocarditis; intracerebral hemorrhage, transient ischemic attack and stroke in past medical history; chronic kidney disease (stage IV and V according to the National Kidney Foundation); severe hypo- and hyperthyroidism; active autoimmune disorder; recorded neoplastic process; taking cytostatic drugs, antiretroviral drugs, glucocorticosteroids, and immunosuppressants; blood transfusion within the last 6 months; documented bone marrow or other transplanted organs; active systemic infection; human immunodeficiency virus (HIV), hepatitis B virus (HBV), or hepatitis C virus (HCV) carrier or positive for hepatitis B surface antigen (HBsAg) or antibodies to HCV; alcohol and drug abuse; pregnancy and lactation; severe injury or surgery in the last month; inability of the patient to collaborate and/or provide informed consent to participate in the study; physical disability preventing the performance of a cardiopulmonary exercise testing; and subjects who did not express their informed consent to participate in a research study.

The research was accepted by the Polish Mother’s Memorial Hospital Research Institute (PMMHRI-BCO.75/2020), and it is in compliance with the Declaration of Helsinki.

### 2.2. Laboratory Tests

Blood samples were collected into polyethylene-terephthalate tubes from each patient. Laboratory tests were obtained following a minimum of 12 h after the last meal in the hospital laboratory. We measured renal function (creatinine, glomerular filtration rate (GFR) was estimated by Modification of Diet in Renal Disease (MDRD)) parameters and liver function (aspartate transaminase (ASP) and alanine aminotransferase (ALT)) parameters; lipoprotein profile: low-density lipoprotein (LDL), high-density lipoprotein (HDL), triglycerides (TG), and total cholesterol (TC); glucose levels; inflammatory cytokine (C-reactive protein (CRP)); and hematology. Additionally, the measurement of N-terminal pro B-type natriuretic peptide (NT-proBNP) and high-sensitivity cardiac troponin T (hs-cTnT) was performed.

### 2.3. Echocardiography

Echocardiograms were performed using the Vivid E95 system (GE Healthcare, Chicago, IL, USA). Quantitative measures were achieved in accordance with current guidelines [15]. The modified biplane Simpson’s rule was necessary to measure left ventricular (LV) volume and ejection fraction (EF). Left atrial (LA) volume was estimated using the modified biplane Simpson’s method from apical 2- and 4-chamber views at end-systole and was indexed to body surface area (LA volume index–LAVi) [16]. Other relevant analyzed parameters were maximal early (E) and late (A) transmitral velocities and the ratio of early to late diastolic transmitral flow velocity (E/A) [17]. We also assessed global peak systolic strain (GLPS) based on speckle tracking echocardiography. The right ventricular (RV) functional measures were tissue Doppler echocardiography (TDE) and tricuspid annular plane systolic excursion (TAPSE) [18].

### 2.4. Spiroergometry

The MetaSoft Studio application software from CORTEX systems was used for CPET. Subjects exercised by cycle ergometer Bike M (CORTEX Biophysik GmbH, Leipzig, Germany) with a metabolic gas analyzer METALYZER 3B (CORTEX Biophysik GmbH, Leipzig, Germany) [19]. Spirometry assessment was conducted before the activity. Forced expiratory volume in one second (FEV1), forced vital capacity (FVC), and FEV1/FVC were measured. Additionally, we evaluated the forced expiratory flow over the middle of one half of the FVC (FEF 25–75). During exercise on a bicycle ergometer, oxygen saturation, electrocardiogram (ECG), blood pressure, and heart rate were monitored. Oxygen uptake is the necessary parameter in the interpretation of CPET. Oxygen uptake (VO_2_) is calculated from the difference between the volume of O_2_ in the inhaled and exhaled air during exercise per unit of time and when the steady state is equal to the metabolic O_2_ consumption. The definition of the VO_2_ peak is the highest attainable VO_2_ for a subject. Other measured CPET parameters include oxygen uptake at anaerobic threshold (VO_2_AT), the minute ventilation/carbon dioxide production slope (VE/VCO_2_ slope), and the respiratory exchange ratio (RER) [20].

### 2.5. Body Mass Analysis

The Segmental Body Composition Analyzer (Tanita Pro, Tokyo, Japan) is a tool to assess non-invasive body mass analysis. This equipment provides estimated values for each measured value using the dual-energy X-ray absorptiometry (DXA) method, estimated value for the total body water measured value by the dilution method and estimated value for the visceral fat using the Bioelectrical Impedance Analysis (BIA method). Subjects were asked to stand barefoot in a stable position. The device provided separate body mass readings for different segments of the body—legs, arms, and whole body—using an algorithm incorporating impedance, age, and height to estimate total and regional fat mass (FM) and fat-free mass (FFM) [21]. The following parameters were also obtained: extracellular water (ECW), intracellular water (ICW), and total body water (TBW). ECW pertains to all body fluid outside the cells. The ICW compartment is the system that includes all fluid enclosed in cells by their plasma membranes. TBW is the total amount of fluid in a person’s body expressed as a percentage of their total weight. Additionally, we calculated the ECW/TBW% ratio [22].

### 2.6. Statistical Analysis

The analysis was obtained using the STATISTICA 13.1 software package (StatSoft, Cracow, Poland). The Shapiro–Wilk test assessed the normality of distribution. To compare two groups, the Student’s *t*-test for continuous variables with normal distribution and Mann-Whitney U test for non-normally distributed variables were used. These categorical data were tested by backward stepwise multivariate logistic regression. In analyses, a *p*-value < 0.05 was considered statistically significant.

## 3. Results

### 3.1. Evaluation of Basic Characteristics

We included 120 patients in this study. The subjects were divided into a group that demonstrated worse oxygen uptake (%VO_2_pred < 80%; *n* = 47) at the median age 49 (IQR: 30–65) and a control group at the median age 55 (IQR: 47–64) who presented with VO_2_pred ≥ 80% (73 patients). The differences between age, height, body mass, body mass index (BMI), and body surface area (BSA) were not statistically significant between groups. Data are presented in Table 1.

### 3.2. Evaluation of Laboratory Tests

Subjects with %VO_2_pred < 80% presented decreased levels of total cholesterol [163.83 (±39.12) vs. 179.00 (±36.22) mg/dL, *p* = 0.03] in comparison to the control group. Statistically significant differences were not observed regarding other biochemical parameters. The results are shown in Table 1.

### 3.3. Evaluation of Echocardiography

**Figure 1:**
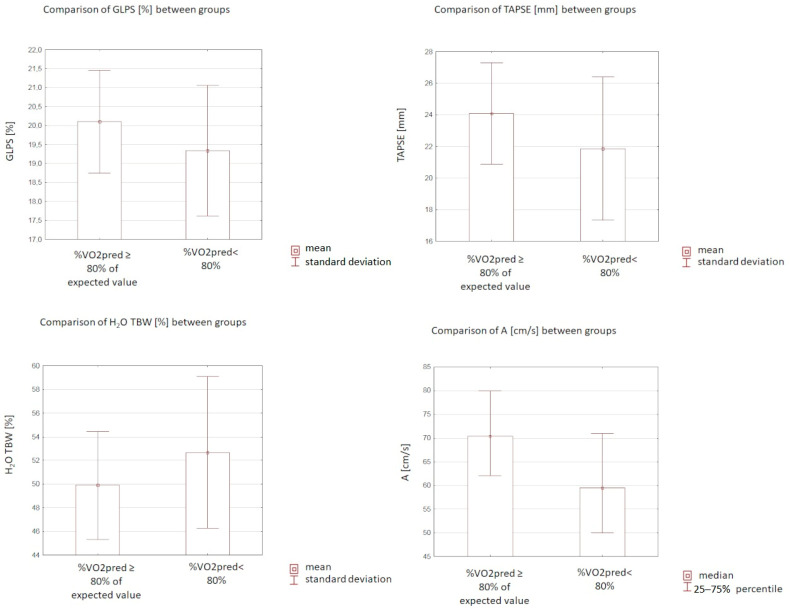
Comparison of selected echocardiographic and body mass analysis parameters between the investigated groups. %VO_2_ pred—percentage predicted oxygen uptake at peak exercise; GLPS—global peak systolic strain; TAPSE—tricuspid annular plane systolic excursion; A—late diastolic filling velocity; H_2_O TBW—total body water.

The (A) velocity, GLPS, and TAPSE were significantly lower (median 59.5 (IQR: 50.0–71.0) vs. 70.5 (IQR: 62.0–80.0) cm/s, *p* = 0.004; 19.34 (±1.72)%vs. 20.10 (±1.35)%, *p* = 0.03; 21.86 (±4.53) vs. 24.08 (±3.2) mm, *p* = 0.002; respectively) in patients who presented with peak VO_2_ < 80% compared to controls. In the study group, E/A was higher (median 1.23 (IQR: 0.98–1.70) vs. 1.01 (IQR: 0.80–1.22), *p* = 0.006) than in the control group. There were no statistically significant differences regarding EF, LA volume, LAVi, E, and TDE S’ (*p* = 0.44; *p* = 0.2; *p* = 0.32; *p* = 0.59; *p* = 0.17; respectively). Data are presented in Table 1 and Figure 1.

### 3.4. Evaluation of Spiroergometry

Patients with worse oxygen consumption presented with significantly decreased HR max, peripheral SBP max, FVC%, and VO_2_AT (132.87 (±33.61) vs. 146.63 (±20.46), *p* = 0.006; median 150 (IQR: 130–170) vs. 180 (IQR: 150–200) mmHg, *p* < 0.0001; 99.61 (±14.74) vs. 111.71 (±16.95) %, *p* < 0.0001; median 12 (IQR: 10–15) vs. 15 (IQR: 13–16) mL/min/kg, *p* = 0.001; respectively). In the study group, the FEF 25–75 was higher in comparison to the controls (3.25 (±1.23) vs. 2.66 (±1.13) L/s, *p* = 0.01). There were no significant differences concerning exercise time, peripheral DBP max, FEV1, FVC (L), FEV1/FVC, FEV1/FVC%, RER, or VE/VCO_2_ slope (*p* = 0.06; *p* = 0.29; *p* = 0.79; *p* = 0.62; *p* = 0.78; *p* = 0.57; *p* = 0.14; *p* = 0.54; respectively). The results are presented in Table 2.

### 3.5. Evaluation of Body Mass Analysis

Considering body mass compartments, only the TBW level (%) was significantly larger in patients with peak VO_2_ ≥ 80% VO_2_ predicted (52.67 (±6.41)% vs. 49.89 (±4.59)%, *p* = 0.02) compared to controls. Statistically significant differences in the remaining parameters (Fat (kg and %), FFM, TBW (kg), ECW, ICW, ECW/TBW) were not detected (*p* = 0.1; *p* = 0.1; *p* = 0.82; *p* = 0.84; *p* = 0.96; *p* = 0.8; *p* = 0.34; respectively). The data are shown in Table 2 and Figure 1.

### 3.6. Multivariate Analysis

Parameters with a *p* value < 0.05 in the univariate analysis were entered into the multivariate analysis using the logistic regression analysis. In a multiple logistic regression model, the two factors were found to be significantly associated with %VO_2_pred were as follows: (A) velocity (OR 0.4, 95%CI: 0.17–0.95; *p* = 0.03) and gender (OR 2.52, 95%CI: 1.07–5.91; *p* = 0.03).

## 4. Discussion

As far as we are aware, the present study is the first analysis of any connection between echocardiographic parameters, hydration status, and worse oxygen uptake in CPET in COVID-19 survivors during Long-COVID. Patients with %VO_2_pred < 80% presented with significantly lower GLPS, TAPSE, and (A) velocity in comparison to controls. A indicates late diastolic mitral flow due to atrial contraction. Peak (A) velocity is often considered a measure of LA function with normal values of 0.8 ± 0.2 m/s in young healthy individuals. Multiple studies have used this parameter as an index of LA function assessment [23]. The percent of TBW content was significantly higher in the study group compared to patients with %VO_2_pred ≥ 80%. The results of multiple logistic regression models independently associated with %VO_2_pred were (A) velocity and male gender.

Oxygen consumption (VO_2_), in addition to physical fitness levels measurement, is an indicator of disease severity in patients with heart failure, chronic obstructive pulmonary disease (COPD), restrictive pulmonary disease, and pulmonary hypertension [24]. Exercise intolerance is defined as an abnormally low VO_2_. Viral toxicity, systemic inflammatory response, changes in the immune system, microvascular injury, fibroblast proliferation due to diffuse alveolar damage, medications, prolonged hospitalization, and stress are considered to be main hypotheses of reduced exercise capacity [25]. Several studies suggested that exercise intolerance could result from physical deconditioning. Motiejunaite et al. conducted a study in which 114 subjects after COVID-19 underwent CPET. During CPET, 75% of the patients had exercise impairment with decreased peak VO_2_ values. The median peak VO_2_ was 17.9 [26]. Decreased exercise tolerance might be associated with mitochondrial injury. Consequently, this phenomenon can lead to reduced energy production during cellular respiration for ATP formation.

The study of Baratto et al. revealed that COVID-19 patients at the time of hospital discharge presented with decreased arterial O_2_ content, higher cardiac output (CO) at rest, and a lower arteriovenous O_2_ difference compared to healthy controls. Additionally, in the study group, a lower muscle O_2_ extraction in the absence of increased pulmonary artery pressure and pulmonary vascular resistance, justifying the reduced peak VO_2_ during exercise, was observed [27]. Some preliminary research studies evaluated exercise tolerance and cardiopulmonary function in ambulatory patients with Long-COVID. Jimeno-Almazàn et al. investigated the association between spiroergometry parameters, echocardiographic parameters, and the severity of symptoms. The study showed that greater exercise tolerance was related to less severe dyspnea and fatigue. Persistent symptoms of Long-COVID were connected with worse fitness level [28]. In one randomized control trial, the authors conducted CPET in 39 participants with Long-COVID. The subjects were randomly divided into a group used to evaluate a tailored exercise program and the control group, which followed the WHO guidelines for rehabilitation after COVID-19. In both groups, improvement of the exercise capacity was observed. Additionally, the authors demonstrated that supervised, tailored exercise programs are more safe and effective in these cases [29]. Echocardiography is readily accessible and may be used to assess for functional cardiac injury. Our findings demonstrated that lower (A) velocity, TAPSE, and GLPS values are related to worse exercise capacity. Previous authors have considered the impact of COVID-19 on the cardiovascular system using transthoracic echocardiography. Tangen et al. enrolled 92 subjects hospitalized in Norway and assessed a TTE three months after infection [30]. All participants had preserved LVEF. A total of 6.5% of the patients presented reduced left ventricle GLS, with no other explanation. A total of 20% of the patients were hospitalized in the intensive care unit, three of them required mechanical ventilation. Another study with 80 adult participants with preserved LVEF demonstrated that 63% of the subjects had symptoms three months after recovery. A total of 25% presented with a decreased GLS, and 8% had a reduced RV GLS [31]. The meta-analysis of Tian et al. showed that lower TAPSE relates to poor COVID-19 disease outcomes [32]. Sex differences have been demonstrated in the acute phase of COVID-19. Males were found to be more vulnerable to developing a severe disease than females, but few studies have assessed sex differences in Long-COVID syndrome. Pelà et al. demonstrated that females were more symptomatic than males, not only in the acute phase but also at follow-up [33]. In another research study, the authors evaluated the predictors of Long-COVID in patients without comorbidities and observed significant differences relating to sex between women with Long-COVID and women without any symptoms after SARS-CoV-2 recovery [34]. However, there have been no studies until now accessing objectively exercise tolerance after COVID-19. In our study, males had a higher risk of persistent limited exercise tolerance. BIA has been suggested as a simple, rapid method to assess changes in hydration status. One of the measured parameters is TBW, which consists of intracellular and extracellular water. In the study of Cornejo-Pareja et al., the authors discovered that overhydration was an important factor of COVID-19 mortality. The researchers enrolled 127 COVID-19 patients. In multivariate analysis, HR was 2.967 (95%CI, 1.459–6.032, *p* = 0.001) for hydration and 2.528 (95%CI, 1.664–3.843, *p* = 0.001) for ECW/TBW [35].

The strength of our study is that this is the first study to assess the etiological and pathophysiological factors influencing the mechanism of exercise intolerance assessed with spiroergometry, as well as the biochemical and echocardiographic parameters in the COVID-19 survivors. We are the first to demonstrate that the patients during Long-COVID without heart failure diagnosis, but with impaired exercise tolerance had worse function of the right ventricle, lower GLPS, LA function, and higher TBW. These findings, however, must be also seen in light of some limitations, including a relatively small study population (120 participants). It needs to be emphasized that it is an ongoing project, and these results need to be considered as preliminary. The study design was limited with regard to the evaluation of the possible effect of used medications. Furthermore, the study included only subjects who could perform CPET. In addition, transthoracic echocardiogram was assessed only at rest. Some echocardiographic parameters, such as left atrial strain, were not evaluated.

## 5. Conclusions

In conclusion, men have over twice the risk of persistent limited exercise tolerance after COVID-19 infection than women. Decreased (A) velocity, TAPSE, and worse GLPS and hydration status are associated with exercise intolerance after COVID-19 in patients with normal LVEF. Further studies are necessary to confirm our results.

## Figures and Tables

**Table 1:** Evaluation of basic characteristics, laboratory tests, and echocardiography among the investigated groups.

Parameter	Peak VO_2_ < 80% VO_2_ Predicted *n* = 47	Peak VO_2_ ≥ 80% VO_2_ Predicted *n* = 73	*p*
Age	(30–65), 49 *	(47–64), 55 *	0.08
Height (cm)	(164–176), 170 *	(164–173), 169 *	0.39
Body mass (kg)	(65–89), 75 *	(69–89), 77 *	0.51
BMI (kg/m^2^)	(22.70–29.98), 25.97 *	(24.61–30.49), 27.28 *	0.07
BSA (m^2^)	(1.69–2.04), 1.86 *	(1.75–2.01), 1.85 *	0.94
hs-cTnT (pg/mL)	(3.70–9.85), 4.90 *	(3.0–6.9), 4.6 *	0.14
NT-proBNP (pg/mL)	(31–125), 73 *	(39–106), 73 *	0.48
Hemoglobin (g/dL)	(12.9–14.6), 13.9 *	(12.4–14.3), 13.3 *	0.11
Creatinine (mg/dL)	(0.65–0.90), 0.78 *	(0.66–0.88), 0.73 *	0.27
GFR (mL/min/1.73 m^2^)	(79.9–107.7), 98.1 *	(79.0–98.7), 89.9 *	0.07
Glucose (mg/dL)	(86–99), 91 *	(86–93), 91 *	0.44
HDL cholesterol (mg/dL)	(35–58), 49 *	(42–59), 50 *	0.38
LDL cholesterol (mg/dL)	92.29 (±34.64)	100.00 (±29.72)	0.20
Triglycerides (mg/dL)	(76–164), 101 *	(90–158), 114 *	0.28
Total cholesterol (mg/dL)	163.83 (±39.12)	179.00 (±36.22)	0.03
ALT (U/L)	(15–25), 22 *	(17–35), 22 *	0.25
AST (U/L)	(24–29), 27 *	(24–32), 26 *	0.47
CRP (mg/L)	(0.5–0.5), 0.5 *	(0.5–0.5), 0.5 *	0.66
EF (%)	(55–66), 62 *	(59–66), 62 *	0.44
LA volume (mL)	(39.0–78.5), 52.0 *	(49–78), 61 *	0.20
LAVi (mL/m^2^)	(21.9–39.5), 30.0 *	(27.0–39.3), 31.9 *	0.32
E (cm/s)	(61–87), 72 *	(60–83), 72 *	0.59
A (cm/s)	(50.0–71.0), 59.5 *	(62.0–80.0), 70.5 *	0.004
E/A	(0.98–1.70), 1.23 *	(0.80–1.22), 1.01 *	0.006
GLPS (%)	19.34 (±1.72)	20.10 (±1.35)	0.03
TAPSE (mm)	21.86 (±4.53)	24.08 (±3.2)	0.002
TDE S’ (cm/s)	(11–15), 14 *	(12–16), 14 *	0.17

*—median; values with non-normal distributions are expressed as median (range) values. Values with normal distributions are expressed as mean ± standard deviation (SD). Peak VO_2_—highest oxygen uptake (VO_2_) during the maximal exercise; VO_2_ predicted—percent predicted oxygen uptake at peak exercise; BMI—body mass index; BSA—body surface area; hs-cTnT—high-sensitivity cardiac troponin; NT-proBNP—N-terminal prohormone of brain natriuretic peptide; GFR—glomerular filtration rate; HDL—high-density lipoprotein; LDL—low-density lipoprotein; ALT—alanine aminotransferase; AST—aspartate aminotransferase; CRP—c-reactive protein; EF—left ventricular ejection fraction; LA—left atrium; LAVi—left atrial volume index; E—early diastolic filling velocity, A—late diastolic filling velocity; E/A—ratio of early to late diastolic transmitral flow velocity; GLPS—global peak systolic strain; TAPSE—tricuspid annular plane systolic excursion; TDE S’—tissue Doppler echocardiography.

**Table 2:** Evaluation of spiroergometry and body mass analysis among the investigated groups.

Parameter	Peak VO_2_ < 80% VO_2_ Predicted *n* = 47	Peak VO_2_ ≥ 80% VO_2_ Predicted *n* = 73	*p*
Exercise time (s)	(378–642), 507 *	(439–697), 580 *	0.06
HR max	132.87 (±33.61)	146.63 (±20.46)	0.006
Peripheral SBP max (mmHg)	(130–170), 150 *	(150–200), 180 *	<0.0001
Peripheral DBP max (mmHg)	(70–90), 80 *	(80–90), 80 *	0.29
FEV1 (L)	(2.55–3.64), 2.99 *	(2.59–3.54), 3.03 *	0.79
FVC (L)	3.90 (±1.05)	3.80 (±0.94)	0.62
FVC%	99.61 (±14.74)	111.71 (±16.95)	<0.0001
FEV1/FVC	(76.0–87.0), 82.5 *	(77–86), 83 *	0.78
FEV1/FVC%	(96–110), 104 *	(97–110), 105 *	0.57
FEF 25–75 (L/s)	3.25 (±1.23)	2.66 (±1.13)	0.01
RER	(1.01–1.10), 1.08 *	(1.03–1.12), 1.09 *	0.14
VO_2max_ (mL/min/kg)	(14–25), 17 *	(20–26), 23 *	<0.0001
VO_2_AT (mL/min/kg)	(10–15), 12 *	(13–16), 15 *	0.001
Peak VO_2max_ (L)	(0.98–1.71), 1.29 *	(1.42–2.08), 1.73 *	<0.0001
VE/VCO_2_ slope	(26.2–34.6), 29.6 *	(25.5–32.6), 29.5 *	0.54
Fat (%)	28.46 (±7.75)	30.92 (±5.47)	0.1
Fat (kg)	(15.7–28.4), 20.5 *	(18.6–29.8), 25.3 *	0.1
FFM (kg)	(47–59.1), 56.5 *	(47.3–63.5), 52.7 *	0.82
TBW (kg)	(34.2–44.0), 41.4 *	(33.7–45.3), 38.5 *	0.84
TBW (%)	52.67 (±6.41)	49.89 (±4.59)	0.02
ECW (kg)	(15.8–19.3), 18.0 *	(14.7–19.8), 17.2 *	(0.96)
ICW (kg)	(19.4–25.5), 24.1 *	(19.1–26.2), 21.7 *	0.8
ECW/TBW × 100%	43.17 (±3.23)	43.77 (±2.37)	0.34

*—median; values with non-normal distributions are expressed as median (range) values. Values with normal distributions are expressed as mean ± standard deviation (SD). Peak VO_2_—highest oxygen uptake (VO_2_) during the maximal exercise; VO_2_ predicted—percent predicted oxygen uptake at peak exercise; DBP—diastolic blood pressure; SBP—systolic blood pressure; FEV1—forced expiratory volume in one second; FVC—forced vital capacity; FEV1/FVC—ratio of forced expiratory volume in one second to forced vital capacity; FEF 25–75%—forced expiratory flow over the middle one half of the FVC; RER—respiratory exchange ratio; VO_2max_—the maximum rate of oxygen consumption attainable during physical exertion per kilogram; VO_2_AT—oxygen uptake at anaerobic threshold per kilogram; peak VO_2_—highest respiratory oxygen uptake (VO_2_) achieved by the subject during the maximal exercise; VE/VCO_2_ slope—the minute ventilation/carbon dioxide production slope; FFM—fat-free body mass; TBW—total body water; ECW—extracellular water; ICW—intracellular water, ECW/TBW%—ratio of extracellular water to total body water.

## Data Availability

Individual participant data that underlie the results reported in this article after deidentification (text, tables, figures, and appendices) as well as study protocol will be available for researchers who provide a methodologically sound proposal. Proposals may be submitted after 9 months and up to 36 months following the article’s publication.
